# Supplementary figures and images for: Assessing the impact of the COVID-19 pandemic on uptake and experiences of gestational diabetes mellitus screening in Ontario: A parallel convergent mixed-methods study
Source: PLoS One. 2024 Dec 27;19(12):e0315983. doi: 10.1371/journal.pone.0315983 (PMC11676491; doi:10.1371/journal.pone.0315983)

**S2 File:** Timeline of lookback windows for outcomes of interest, by birth group

**
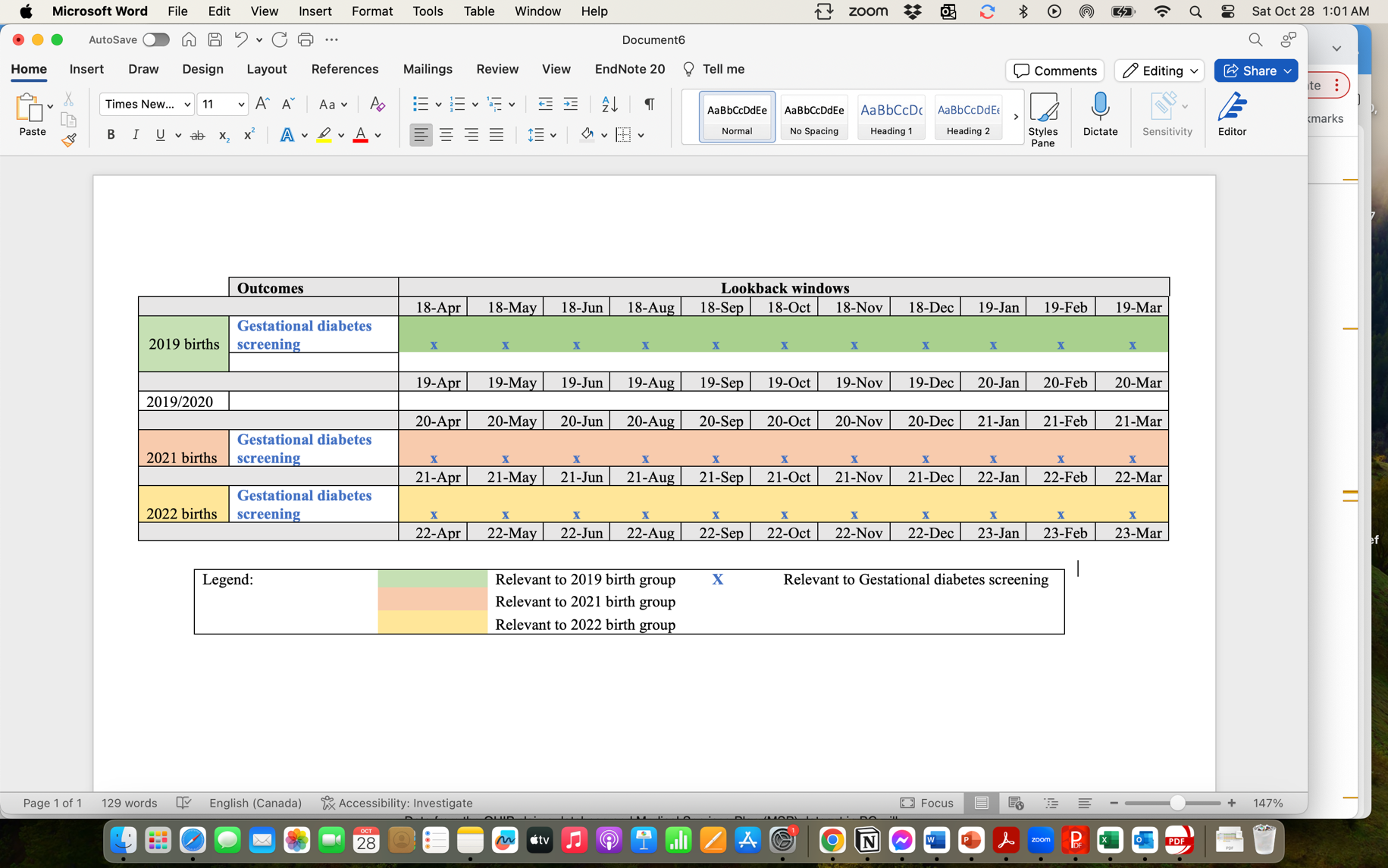
**

Supplement: S2 File — (DOCX) [file pone.0315983.s002.docx]
