# Supplementary material for: Assessing the impact of the COVID-19 pandemic on uptake and experiences of gestational diabetes mellitus screening in Ontario: A parallel convergent mixed-methods study
Source: PLoS One. 2024 Dec 27;19(12):e0315983. doi: 10.1371/journal.pone.0315983 (PMC11676491; doi:10.1371/journal.pone.0315983)
Supplement: S3 File — (DOCX) [file pone.0315983.s003.docx]

**S3 File:** Description of data source

| **Dataset** | **Description** | **Intended Purpose** |
| --- | --- | --- |
|  |  |  |
| Ontario Mother-Baby linked dataset (MOMBABY) | MOMBABY includes all inpatient birth admission records from the Discharge Abstract Database. It links mothers and their newborns deterministically based on the maternal/newborn chart number. | - Cohort creation (i.e., to identify pregnant persons and newborns) - Baseline characteristics (e.g., parity) |
| Ontario Health Insurance Policy (OHIP) | The OHIP claims database contains information on publicly funded services, primarily provided by physicians, to Ontario residents eligible for the health insurance system. The main data elements are service/billing codes for the service provided. | - Covariates and confounders of interest (e.g., perinatal care provider type, prenatal care visit count^[[1]](#footnote-1)^) |
| Ontario Diabetes Database (ODD) | ODD is a validated registry of all people in Ontario diagnosed with diabetes | - Cohort creation (i.e., excluding participants with pre-existing diabetes)^[[2]](#footnote-2)^ |
| Canadian Index of Multiple Deprivation (CIMD) | The CIMD is a geographically based index developed to quantify the degree of marginalization occurring across the country using census data. | - Baseline characteristics (e.g., ethnocultural composition, residential instability, economic dependency) |
| Registered Persons Database (RPDB) | The RPDB contains basic demographic information about anyone who has ever received an Ontario health card number. | - Baseline characteristics (e.g., maternal age) |
| Ontario Laboratory Information System (OLIS) | OLIS provided lab results of patients from all public Health Ontario laboratories, including hospitals and community laboratories. | Outcome definition: Gestational diabetes screening ^[[3]](#footnote-3)^ |

1. The type of perinatal care provider for each participant was determined based on the specialty of the provider who had the most Ontario Health Insurance Plan (OHIP) billings for P004 (minor prenatal assessment) during the period between the participant's conception and birthing dates. In cases where there was an equal number of P004 fee codes billed by two different types of providers (for example, family physicians and obstetricians), the care was categorized as "shared." Identification of midwifery clients was based on one of two criteria: either the service provider number was "11004" in the Discharge Abstract Database (DAD) hospital record, or any of the following OHIP fee codes were billed between the conception and birthing dates: A800, A801, A802, A813, A815, A816, C800, C801, C802, C813, C815, C816, K224, or Q513. [↑](#footnote-ref-1)
2. Those with pre-existing diabetes were identified with the following code: DIAG_LAST. [↑](#footnote-ref-2)
3. **HbA1c test (Hemoglobin A1c):** HbA1c (in pregnancy) was used consistently with OLIS and included LOINC codes 17855-8, 17856-6, 41995-2, 4548-4, 59261-8, and 71875-9. **50 g glucose challenge test:** LOINC codes 14754-6 or 14756-1 were used, specifically when the same observation date coincided with an observation using LOINC 4269-7 with a value of 50, or when LOINC 14756-1 was used in cases where no observation (or an observation with a missing value) with LOINC 4269-7 on the same observation date and there were no other glucose tests conducted on the same observation date. **75 g oral glucose tolerance test:** LOINC codes L104 or L103 were used. L103 was specifically designated for GTT in pregnancy. This test involved a 75 g oral glucose tolerance test for the diagnosis of gestational diabetes. It necessitated separate observations for fasting, 1-hour, and 2-hour values on the same ordersid. Potential LOINC codes for the fasting value included 1552-9, 14749-6, 14771-0, 14996-3, 15074-8, 39480-9, and 47622-6. Potential LOINC codes for the 1-hour value included 14756-1 and 51597-3, while potential LOINC codes for the 2-hour value included 14759-5 and 14995-5. In addition, at least one of the following conditions needed to be met: the observation for the fasting value had LOINC 1552-9 or 14996-3, the observation for the 1-hour value had LOINC 51597-3, the observation for the 2-hour value had LOINC 14995-5, or the same ordersid also had an observation for LOINC 4269-7 with a value of 75. **Non fasting random plasma glucose test:** LOINC codes 14749-6, 15074-8, and 39480-9 were used. **Timing of GDM screening**: List of LOINC codes: 1552-9, 4269-7, 14749-6, 14754-6, 14756-1, 14759-5, 14771-0, 14995-5, 14996-3, 15074-8, 39480-9, 47622-6, 51597-3 [↑](#footnote-ref-3)
