# Supplementary material for: Assessing the impact of the COVID-19 pandemic on uptake and experiences of gestational diabetes mellitus screening in Ontario: A parallel convergent mixed-methods study
Source: PLoS One. 2024 Dec 27;19(12):e0315983. doi: 10.1371/journal.pone.0315983 (PMC11676491; doi:10.1371/journal.pone.0315983)
Supplement: S4 File — (DOCX) [file pone.0315983.s004.docx]

**S4 File**: Qualitative interview guide

**Introduction to the study**

Our study is interested in how your perception of how COVID-19 risk influenced the decisions you made about pregnancy in three different areas: prenatal care, vaccination, and social support. We know that those decisions are related to so many other things in life, so I’m going to start by asking a few questions about what your life was like at the time you became pregnant, and how the pandemic affected you. Then we will move into some specific questions about those three areas: prenatal care, vaccination, and social support. If I ask any questions you don’t wish to answer, you are welcome to skip that question, and you can also end our interview at any time if you need to do so.

These interviews are really about understanding your thoughts, opinions and beliefs. There is no right or wrong answer. Please feel free to give us details, tell stories, or talk about your experiences in depth.

**Informed consent script**

Did you receive and review a copy of the informed consent letter?

Did you have the opportunity to ask questions and were they answered to your satisfaction?

Do you understand that you have the right not to participate and the right to withdraw at any time?

Do you understand that any information you provide will be kept confidential, unless required by law, and that only the study researchers and staff involved in this study, independent ethics committees and inspectors from government regulatory agencies will have direct access to the information you provide?

And finally, do you consent to participating in this recorded interview?

**INTRODUCTION**

1. Just to situate us within the pandemic, do you remember what month and year it was when you first found out you were pregnant?
   1. And when did you give birth?
2. Could you tell me a bit about what your life was like right around the time you found out you were pregnant? Who did you live with, how did you spend your time, that kind of thing.
   1. Employed? Doing what kind of work?
   2. Partner/family situation?
3. Tell me about what the pandemic was like when you realized you were pregnant?
   1. Public health restrictions such as closures, curfews, mask requirements, or vaccine passports?
   2. What was your work situation like?
   3. Vaccines available?
   4. Had you or anyone in your social sphere had COVID-19?
4. We know that many pregnancies are not planned, but that doesn’t mean they’re not wanted. On the spectrum of very carefully planned to a complete surprise, where was this pregnancy?
   1. If planned- how did the pandemic influence your decision to try and have a baby?
   2. If not-so-planned, how did the pandemic influence your reaction to finding out you were pregnant?
5. Is this your first child?
   1. If no, how many others live in your household? How old were they when this child was born?
   2. If yes, are there any other children in your household or for whom you provide co-parenting or care, such as step-children or younger relatives?

**RISK PERCEPTION**

1. Thinking back to when you were pregnant, on a scale of 1-10, how concerned were you about COVID, and why? (1 being not concerned at all, and 10 being extremely concerned)

How did your level of concern impact what you did or didn’t do to try to avoid infection?

Did your level of concern change during your pregnancy? *(Probe: before, during, and after)*

You rated your level of concern as a ________ *(insert number).* How does this number reflect your concern about you and baby, your household, your network, and your community?

What were the sources of information that influenced your level of concern? *(Probe: public health, friends, family, social media, practitioners)*

**VIRTUAL CARE**

Virtual care is any form of health care service not delivered in person. This could be over the phone, or on a video call, anytime you met with a healthcare provider like a doctor, lactation consultant, but weren’t physically present in the same location.

1. During your pregnancy, did you have any virtual visits? *If no, skip this section.*
   1. Could you tell me what kind of visits did you have virtually, and what did you think of that?
   2. Did you get to choose if you had a virtual visit?
      1. What did you like?
      2. What didn’t you like?
      3. What would you change?
   3. After the pandemic is over, what kind of appointments should still be available virtually?

**PRENATAL CARE AND VACCINATION**

1. First I’m going to ask you about some decisions relevant to the type of prenatal care you received. To set the stage, what kind of prenatal care provider or providers did you have?
2. [if multiparous] You told me earlier this wasn’t your first child- how did the pandemic change the way that you interacted with your healthcare provider, compared to before the pandemic?
3. Most people get offered what’s called a glucose challenge test while they are pregnant, in the second trimester, to see if they are at risk of having a particular kind of diabetes that happens during pregnancy. In this test, you drink a very sugary liquid and then wait a few hours and have your blood drawn to see how your body processed the sugar.
   1. Were you offered this test? Was it your decision whether or not to accept it?
      1. Did you choose to do it? What were your considerations while making this decision?
   2. Did your healthcare provider offer an alternative to this test for diabetes?
4. Do you recall being offered or recommended any vaccinations during this pregnancy?
   1. Which ones? (Typical answers: pertussis/whooping cough/third trimester TDaP, seasonal influenza in Oct-April most years; and for 2021 COVID mRNA vaccine)
   2. Did you get them? Why or why not?
5. Very briefly, what is your overall view of vaccines?
   1. How safe and effective do you think the vaccines available to day in Canada are?
   2. How important do you think it is to vaccinate your baby according to the recommended schedule?
   3. And what is your general view of the vaccines available in Canada to protect against COVID-19, specifically?
6. For 2021 births: Were you offered a COVID vaccine during pregnancy? (Skip if they have already said this)
   1. If yes: do you remember when this was, and which dose it was for you?
   2. If no: do you know why not? (No vaccine yet, weren’t eligible where you lived, already vaccinated, etc.)

**IF OFFERED COVID VACCINE DURING PREGNANCY:**

1. How did you decide whether and when to get vaccinated?
   1. What were your main considerations in this decision?
   2. Can you compare this with your decision about getting the TDaP/pertussis vaccine during pregnancy? Were you more comfortable with one over the other? Why?
2. When deciding whether to get the COVID-19 vaccine…
   1. Who did you talk with when making the decision?
   2. What information did you look for?
   3. What did you think of the information you received?
   4. Was there any information you wished you had and didn’t find?
   5. Were there any people or media sources offering advice you wished you could avoid?
   6. How did you decide what information to trust?
   7. Ultimately, what information do you feel was influential or important to your decision?
3. Did you talk about the COVID vaccine with any health care providers?
   1. What did they recommend?
   2. What questions did you have?
   3. Were they able to answer those questions?
   4. Did they provide other information?

## **BIRTH AND POSTPARTUM**

1. Did you get to decide where you were going to give birth?
   1. Where did you plan to give birth?
   2. How did the pandemic influence that decision?
   3. And did you end up giving birth in the place you planned?
2. For hospital births: After you gave birth, how long did you stay in the hospital?
   1. How did the pandemic influence your time in hospital after having your baby?
      1. Probe: visitors or a support person?
   2. Was it your choice about when to go home? What influenced that decision?

**SOCIAL SUPPORT AND MENTAL HEALTH**

1. Let’s talk about your postpartum experience. What were your expectations for the social support you would receive after birth?
   1. If probe needed: help at home, visitors, Baby celebrations
   2. Probe: expectations formed from previous birth? Participation in supporting other new moms etc?
   3. Was that what happened? What was different from your expectations?
   4. In what ways did COVID affect your expectations and experience?
2. During the pandemic, many people had to make tough decisions about limiting their social contacts. How did you handle this challenge while you were pregnant?
3. Was there anyone you wanted to see but chose not to?
4. When someone invited you to do something, like participate in a baby shower, meet up for dinner, or go to a birthday gathering, how did you make that decision?
5. We know that many people experienced depression or anxiety during the pandemic, and that pregnancy can be a particularly vulnerable time of life for feeling depressed or anxious. Did you experience any feelings of depression or anxiety when pregnant or after giving birth?
   1. How did these feelings affect you?
   2. What things did you do to try to deal with these feelings?
   3. Were there things you did differently because you were feeling like this? (For example, done more socializing than might have been ideal for COVID risk.)
   4. Were there people in your life who were particularly helpful or supportive? (What did they do, specifically related to depression and anxiety?)
6. Did you ever consider accessing professional support or any other kind of help for your mental health?

If no,

- 1. Were there factors that contributed to your decision not to seek support (e.g., perception that support wouldn’t be helpful, thought that the symptoms would eventually resolve over time, too costly, stigma, etc.)?

If yes,

- 1. How did you make the decision to seek help? Was anyone else involved in this decision?
  2. Were you able to access help? How did that go?
  3. What type(s) of support did you seek?
  4. How did you decide to pursue [that kind of support]? Probe:(e.g., affordability, convenience, past experiences or relationships, recommendations.)?
  5. Was there anything that was difficult about accessing [this support]?
  6. Was this your first choice of support? Or was there something else that you might have preferred but which was not available or mor difficult to access?
     1. Probe if need (for example because of financial reasons, waitlist, not convenient)
  7. Was this [initial support] what you needed? Did you have to look for something else?

**CLOSING**

1. Those are all the questions I had. Is there anything I didn’t ask you that you think is important for me to know?

That’s the end of our interview. I want to thank you for your time, and thank you for sharing your experiences with me. I know it takes a level of trust to do that and I’m so grateful that you’ve entrusted me with your thoughts, feelings, and experiences. If we have any follow up questions as we work with your interview, would it be ok to re-contact you to ask for more information?

**If yes:** would you prefer us to e-mail or text or phone you?

**If no:** no problem, thanks for considering.

I hope you have a wonderful rest of your day, and if you don’t have any more questions, you are welcome to leave whenever you’re ready.
